# An EMG-Based GRU Model for Estimating Foot Pressure to Support Active Ankle Orthosis Development

**DOI:** 10.3390/s25113558

**Published:** 2025-06-05

**Authors:** Praveen Nuwantha Gunaratne, Hiroki Tamura

**Affiliations:** 1 Interdisciplinary Graduate School of Agriculture and Engineering, University of Miyazaki, 1-1 Gakuen Kibanadai-nishi, Miyazaki 889-2192, Japan; 2 Faculty of Engineering, University of Miyazaki, 1-1 Gakuen Kibanadai-nishi, Miyazaki 889-2192, Japan

**Keywords:** surface EMG, GRU neural network, foot pressure estimation, gait analysis, AAFO

## Abstract

As populations age, particularly in countries like Japan, mobility impairments related to ankle joint dysfunction, such as foot drop, instability, and reduced gait adaptability, have become a significant concern. Active ankle–foot orthoses (AAFO) offer targeted support during walking; however, most existing systems rely on rule-based or threshold-based control, which are often limited to sagittal plane movements and lacking adaptability to subject-specific gait variations. This study proposes an approach driven by neuromuscular activation using surface electromyography (EMG) and a Gated Recurrent Unit (GRU)-based deep learning model to predict plantar pressure distributions at the heel, midfoot, and toe regions during gait. EMG signals were collected from four key ankle muscles, and plantar pressures were recorded using a customized sandal-integrated force-sensitive resistor (FSR) system. The data underwent comprehensive preprocessing and segmentation using a sliding window method. Root mean square (RMS) values were extracted as the primary input feature due to their consistent performance in capturing muscle activation intensity. The GRU model successfully generalized across subjects, enabling the accurate real-time inference of critical gait events such as heel strike, mid-stance, and toe off. This biomechanical evaluation demonstrated strong signal compatibility, while also identifying individual variations in electromechanical delay (EMD). The proposed predictive framework offers a scalable and interpretable approach to improving real-time AAFO control by synchronizing assistance with user-specific gait dynamics.

## 1. Introduction

Japan, like many developed nations, is facing an unprecedented demographic transformation. With over 29% of its population aged 65 years and above, age-related mobility impairments are becoming increasingly prevalent [1]. Among the elderly, lower limb dysfunction, including weakness in ankle dorsiflexion, imbalance, and reduced gait speed, has emerged as a primary contributor to falls, limited mobility, and reduced independence [2,3]. These challenges have created an urgent demand for advanced assistive technologies and targeted rehabilitation solutions capable of addressing an aging society’s complex biomechanical and neuromuscular needs [4]. Among the critical areas of focus, the ankle joint complex plays a key role in enabling a safe, stable, and efficient gait. It acts as a dynamic interface between the body and ground, bearing weight and modulating movement across three anatomical planes: sagittal (dorsiflexion and plantarflexion), frontal (inversion and eversion), and transverse (abduction and adduction) [5]. While sagittal plane dynamics, especially dorsiflexion and plantarflexion, have been the primary focus in both clinical assessments and device control strategies [6], recent studies highlight the essential contribution of frontal and transverse movements in ensuring balance, adaptability to uneven surfaces, and overall gait symmetry [7]. The inability to coordinate these multi-planar movements can result in abnormal gait patterns, poor load distribution, and an increased risk of injury [8].

**Figure 1:**
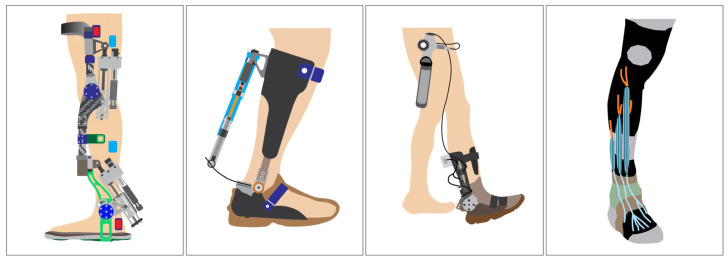
Example AAFO designs proposed by previous researchers featuring integrated sensor–actuator systems for gait event detection and providing ankle joint assistance [9].

In response to these challenges, ankle–foot orthoses (AFO), especially AAFO, have been developed to assist or restore ankle joint function [9,10,11]. These devices use sensors and actuators to detect gait events and provide timely mechanical support (see Figure 1), particularly during dorsiflexion failure as observed in foot drop [12]. Traditional rigid AFO offer joint immobilization and passive correction, while articulated AFO provide limited dynamic control by allowing a partial range of motion [13].

However, both types often restrict natural joint kinematics and fail to deliver phase-specific assistance [14]. To overcome these limitations, modern robotic AAFO integrate active control mechanisms and aim to replicate physiological ankle movement [15]. However, some significant challenges persist in both hardware (e.g., actuator miniaturization, power efficiency, and sensor integration) and control architecture (e.g., real-time adaptability, multi-planar movement estimation, and subject-specific variability) [16,17]. A primary limitation of existing AAFO is the use of simplified control strategies, typically based on fixed thresholds, rule-based algorithms, or pre-programmed assistance limited to sagittal plane movements [18]. These methods are inadequate for addressing complex gait deviations, particularly when multi-directional ankle actions like inversion, eversion, or plantarflexion during push off are involved [19]. Additionally, there is often a lack of robust real-time estimation of foot–ground interaction forces, which is essential for adaptive and intuitive control [20]. Addressing this gap requires a predictive approach that can infer spatial plantar pressure distributions from neuromuscular activity in a dynamic, subject-independent manner.

Several studies have explored advanced control strategies to overcome these limitations. For instance, non-parametric neural network models such as multilayer perceptrons (MLPs) have been applied to model AFO dynamics and estimate ground reaction forces with high accuracy [21,22]. While these systems showed low prediction errors, they primarily focused on mechanical system identification and did not utilize EMG-based neuromuscular inputs. Other research integrated EMG and inertial measurement unit (IMU) data with classifiers and sequential models such as long short-term memory (LSTM) and transformer networks for gait event detection or abnormality classification [23,24]. Despite achieving high accuracy in phase classification, these approaches did not predict continuous, region-specific plantar pressure in real time. Furthermore, data-driven predictive controllers, including model predictive control (MPC) and disturbance rejection strategies, have been proposed for AAFO [25,26], but these designs remain largely reactive and do not incorporate proactive neuromuscular prediction. Collectively, these efforts underscore a significant research gap, namely, the absence of interpretable, temporally aligned models that leverage EMG signals to estimate continuous foot–ground pressure distributions for adaptive AAFO control.

This study proposes a GRU-based deep learning model for predicting plantar pressure values at key foot regions (heel, midfoot, and toe) using surface EMG signals. The model is designed to estimate the temporal relationship between muscle activation patterns and foot pressure dynamics, thereby enabling the real-time inference of gait events and associated ankle joint functions [27]. EMG signals are inherently noisy and exhibit inter-subject variability; therefore, we applied comprehensive signal preprocessing, including band pass filtering, notch filtering, rectification, and normalization [28]. A sliding window approach was used to segment the EMG time series, and the RMS feature was extracted from each window as it consistently demonstrated the strongest correlation with FSR pressure outputs among other time-domain and frequency-domain features [29]. The model architecture is centered on a GRU network, selected for its ability to model temporal dependencies in EMG signals with a lightweight structure suitable for embedded systems. The model was trained using data from multiple subjects and evaluated based on mean squared error (MSE) and mean absolute error (MAE). The time lag between EMG activity and corresponding foot pressure, a common challenge in neuromuscular modeling, was manually addressed during feature alignment. While this method assumes temporal consistency across subjects, the analysis revealed individual variations, underscoring the need for the future development of adaptive alignment strategies [30,31]. This modeling approach is directly applicable to AAFO control systems, enabling real-time pressure prediction to identify gait phases such as initial contact (heel strike), mid-stance, and push off (toe off), each corresponding to specific ankle joint actions [32]. This information can be used to trigger actuator responses for specific gait assistance scenarios, such as compensating for foot drop during the swing phase, enhancing plantarflexion during propulsion, or stabilizing inversion/eversion movements on uneven terrain. Unlike conventional AAFO systems, which rely on mechanical sensing or phase detection algorithms, this EMG-based predictive framework offers a personalized control approach driven by neuromuscular signals [33].

In conclusion, this study presents an interpretable GRU-based model for predicting plantar pressure from EMG signals, addressing key limitations in current AAFO control strategies. While previous studies have utilized combinations of EMG and IMU signals for gait phase classification or joint trajectory estimation [23,24], the present work emphasizes the continuous prediction of spatially distributed plantar pressure patterns using surface EMG signals as the primary input modality. Rather than classifying discrete gait phases, the model captures temporally aligned pressure dynamics at anatomically segmented foot regions, namely the heel, midfoot, and toe, enabling the fine-grained inference of gait events. This temporal modeling approach, driven by neuromuscular activation patterns, offers a physiologically relevant and computationally efficient solution that supports adaptive, multi-phase, and directionally responsive assistance. Accordingly, the proposed framework contributes to the development of personalized AAFO systems that synchronize actuator output with real-time muscle intent, facilitating more natural and effective gait rehabilitation.

## 2. Considerations for Evaluation

### 2.1. Gait Cycle

**Figure 2:**
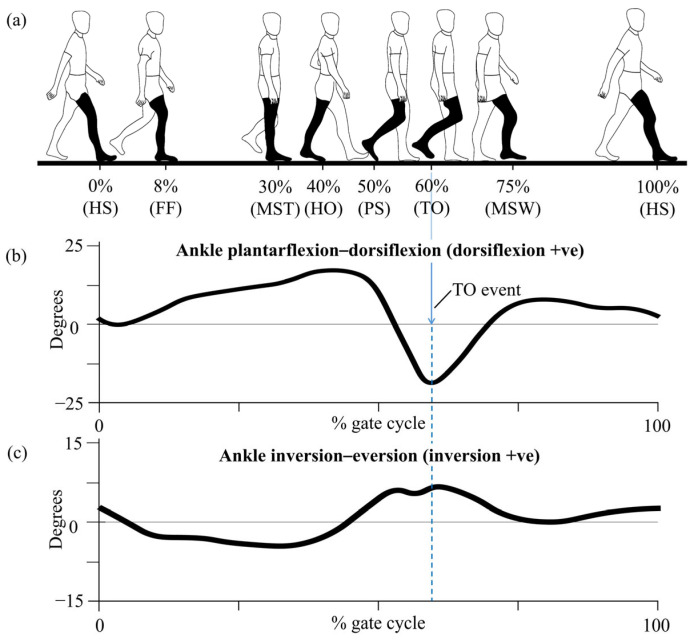
Schematic of the human gait cycle: (**a**) key temporal events including heel strike (HS), foot flat (FF), mid-stance (MST), heel off (HO), pre-swing (PS), toe off (TO), and mid-swing (MSW); (**b**) representative ankle joint movements in the sagittal plane; (**c**) corresponding movements in the frontal plane.

Human walking is characterized by a repetitive, coordinated sequence of lower limb movements collectively known as the gait cycle. A single gait cycle is defined as the time interval between two successive occurrences of an initial heel strike (HS) of the same foot [34]. This cyclical pattern allows the body to move forward in a stable and energy-efficient manner, involving alternating periods of support and limb advancement. Figure 2 presents a schematic representation of the human gait cycle, illustrating key temporal events and associated joint movements across different anatomical planes. The gait cycle is broadly divided into two main phases: the stance phase and the swing phase. The stance phase, which accounts for approximately 60% of the total cycle, begins at HS and ends at toe off (TO), during which the foot remains in contact with the ground. The swing phase follows, occupying the remaining 40%, and represents the period during which the foot is lifted from the ground and progresses forward in preparation for the next HS [3]. Within these two broad phases, the gait cycle can be further segmented into several key gait events, including foot flat (FF), mid-stance (MST), heel off (HO), TO, and terminal swing, each representing important transitional points in lower limb mechanics (see Figure 2a) [35].

In the sagittal plane, the cycle begins at HS, where the ankle is positioned in slight plantarflexion to absorb ground reaction forces. As the foot transitions to an FF position, the ankle moves into dorsiflexion, enabling shock absorption and body weight acceptance. During MST, dorsiflexion continues as the body’s center of mass shifts forward over the supporting foot. Toward the end of the stance phase, the ankle initiates plantarflexion, reaching its peak at the TO event, which generates the propulsive force required for forward progression. During the swing phase, the ankle returns to dorsiflexion to achieve ground clearance and then transitions into slight plantarflexion in preparation for the next HS (see Figure 2b) [36].

Although sagittal plane movements, especially dorsiflexion and plantarflexion, are the most visually evident and frequently analyzed, frontal plane movements such as inversion and eversion are equally critical for maintaining lateral stability [37]. As the foot approaches HS, it typically lands in a slightly inverted position, improving lateral stability upon contact. As the body progresses into MST, the foot transitions into eversion, helping to distribute plantar pressures more evenly and adapt to irregular surfaces, which is vital for balance and preventing falls [38]. Before TO, the foot once again moves into inversion, forming a rigid lever necessary for effective propulsion and efficient energy transfer into the swing phase (see Figure 2c). These controlled mediolateral movements, typically occurring within ±15 degrees, are particularly important in individuals with impaired gait stability [39,40].

Movements in the transverse plane involve subtle internal and external rotations of the foot and ankle, often overshadowed by the more dominant sagittal and frontal plane actions. Though less prominent, transverse plane adjustments are especially relevant in complex tasks such as turning, obstacle negotiation, and adapting to sloped surfaces [41]. From our standpoint, the ability to precisely identify and respond to these key gait events is essential, particularly in the development of intelligent AAFO. The real-time recognition of gait transitions enables orthotic systems to synchronize assistance with user intent and gait phase demands [42]. Detecting TO allows timely plantarflexion support to aid propulsion while identifying HS, which can trigger dorsiflexion assistance to stabilize the ankle during loading. Similarly, understanding MST dynamics can inform balance-oriented corrections through inversion/eversion support, which is especially critical in populations at risk of lateral instability or falls [43]. In the context of this study, this EMG-based GRU model is capable of predicting FSR pressure values at the heel, midfoot, and toe regions—corresponding to critical gait events throughout the walking cycle. These predicted pressure profiles function as real-time indicators of events such as HS, MST, and TO, enabling targeted and phase-specific actuation in AAFO systems [44]. These specific gait events were prioritized in our model design due to their biomechanical significance; each event represents a transition point involving rapid changes in ankle loading or unloading, where timely neuromuscular assistance can meaningfully enhance gait safety, propulsion, and postural stability [45].

### 2.2. Anatomy of the Ankle Joint Complex

**Figure 3:**
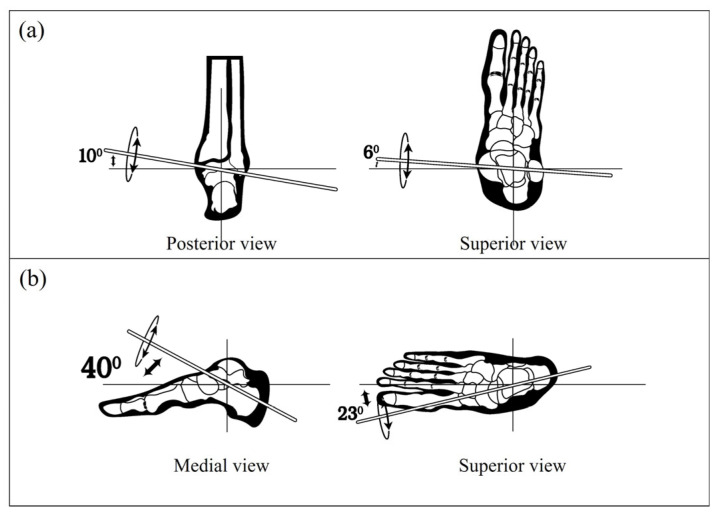
Oblique orientation of the rotational axes in ankle joint complex illustrating their multi-planar contributions to ankle joint motion: (**a**) the talocrural joint; (**b**) the subtalar joint.

The ankle joint complex is fundamental in human locomotion, serving as the dynamic interface between the lower limb and the ground. Structurally, it comprises three primary articulations: the talocrural joint, the subtalar joint, and the transverse-tarsal joint. Each contributes to the intricate multidirectional mobility required for efficient gait and balance. These articulations work in a coordinated fashion to accommodate changes in terrain, absorb impact forces, and maintain postural control functions that are especially critical in developing responsive AAFO [46]. The talocrural joint is traditionally characterized as a hinge type synovial joint formed by the articulation between the distal tibia and fibula with the talus. This joint primarily enables plantarflexion and dorsiflexion key movements in forward propulsion and foot clearance during gait. However, its axis of rotation is not purely aligned with the sagittal plane; instead, it follows an oblique trajectory, slanting slightly downward and laterally (see Figure 3a) [47]. This obliquity introduces subtle components of transverse and frontal plane motion, increasing the joint’s biomechanical complexity and making it more functionally adaptable than a simple hinge would suggest [48].

Inferior to the talocrural joint lies the subtalar joint, which facilitates the inversion and eversion of the foot. This articulation, formed between the talus and calcaneus, also operates around an oblique axis (see Figure 3b) angled medially and anteriorly, which enables it to contribute to compound motions such as pronation and supination [49]. These combined actions are essential for mediolateral stability, particularly during stance phase transitions [50]. The subtalar joint also interacts functionally with the transverse-tarsal joint, comprising the talonavicular and calcaneocuboid joints. The combined mobility of these two joints enhances the foot’s ability to adjust to varying ground surfaces and maintain dynamic equilibrium. This coupling is critical during tasks that require rapid adaptation, such as turning or walking on uneven terrain [51,52].

The complexity of the ankle joint’s mechanical behavior is mirrored in its neuromuscular control architecture. Twelve extrinsic muscles control foot and ankle motions and are categorized into four functional compartments [53]. The anterior compartment includes the tibialis anterior (TA), a key muscle in dorsiflexion and a frequent focus in EMG-based foot drop analysis [54]. The lateral compartment, containing the fibularis longus (FL) and brevis, facilitates plantarflexion and eversion [55]. The posterior compartments are further divided into superficial and deep layers. The superficial posterior compartment, including the gastrocnemius (GA) and soleus (SOL), is primarily responsible for plantarflexion, especially during the push off event that occurs in the pre-swing phase of the gait cycle [56]. The deep posterior compartment, including the tibialis posterior, contributes to inversion and stabilization of the medial arch [57].

This anatomical arrangement reflects not only the mechanical interdependence but also the electromyographic complexity involved in generating and controlling ankle motion. From a modeling perspective, this has direct implications. Since our study utilizes surface EMG signals to estimate plantar pressure distributions, understanding which muscles contribute to which movement directions and how these movements manifest during different gait phases is essential [28]. Moreover, the overlapping functions of certain muscles across multiple planes (e.g., TA in dorsiflexion and inversion) present challenges for signal interpretation and movement differentiation, especially in real-time control systems of AAFO [58,59].

## 3. Methodology

### 3.1. Participants

This study involved primarily a cohort of four healthy male participants (subjects I–IV), all of whom voluntarily participated after providing written informed consent. One additional participant (subject V) with similar demographic characteristics and inclusion criteria was reserved exclusively for test data acquisition to evaluate model generalization performance. The subjects I–V had a mean age of 23.6 ± 1.52 years, mean weight of 60.4 ± 7.09 kg, mean height of 166.6 ± 3.51 cm, and mean BMI of 21.77 ± 2.58 kg/m^2^. Subjects were screened before inclusion to ensure the absence of any musculoskeletal, neurological, or systemic conditions that could affect gait performance. All data were collected from the right leg to maintain consistency, as all participants self-reported right leg dominance. The experimental procedures were conducted in accordance with the ethical standards and protocols approved by Prof. Tamura’s laboratory at the University of Miyazaki.

### 3.2. Experimental Setup

The experimental system was designed to simultaneously collect surface EMG and plantar pressure data during normal walking trials. Two primary sensor platforms were utilized:

- four wireless surface EMG sensors (Trigno, Delsys Inc., Natick, MA, USA);
- a four-channel FSR sensor system using the Delsys Trigno 4-Ch FSR Adapter (Delsys Inc., Natick, MA, USA).

**Figure 4:**
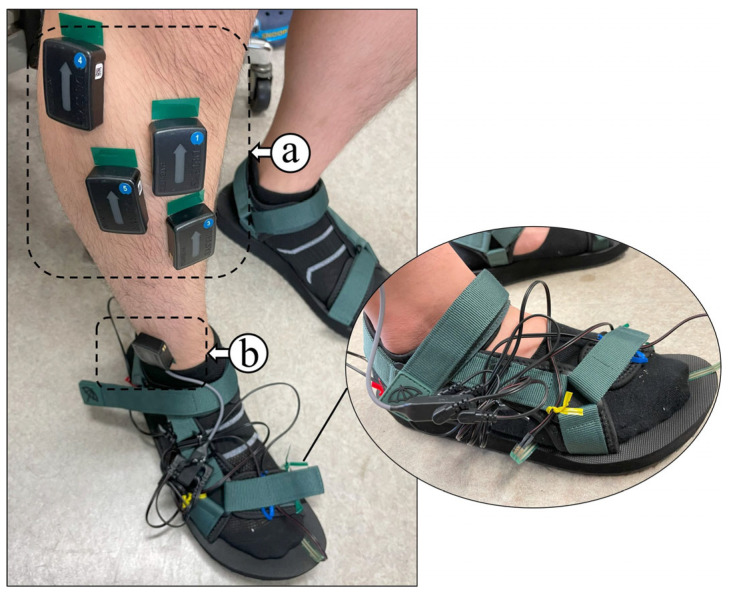
Sensor configuration used for data acquisition: (a) wireless surface EMG sensor placement over target lower limb muscles; (b) wireless FSR sensor unit with attached FSR membrane for plantar pressure measurement.

The surface EMG sensors were positioned over the muscle bellies of four key muscles of the right lower leg: the TA, FL, SOL, and GA. These muscles were selected based on their primary roles in ankle biomechanics, particularly dorsiflexion (TA), eversion and lateral stabilization (FL), and plantarflexion (SOL and GA), all of which are critical during different gait events. Sensor placement was performed by established anatomical landmarks to ensure signal reliability and repeatability across subjects. The sensor configuration used for data collection is shown in Figure 4, including the wireless sEMG and FSR systems.

**Figure 5:**
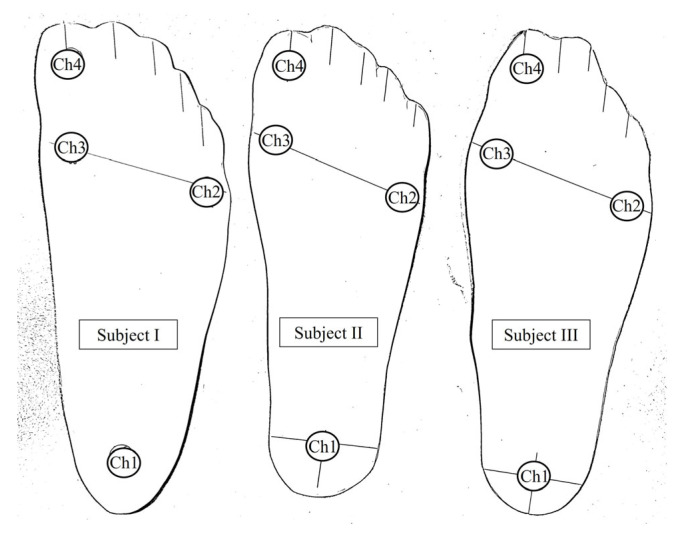
Placement of FSR membranes customized to subject-specific foot geometry, aligned with anatomical pressure zones corresponding to channels 1–4 (heel, midfoot, and toe regions).

Each Delsys surface EMG sensor featured a compact body size of 27 × 37 × 13 mm and a mass of 14 g, offering a lightweight and unobtrusive profile suitable for gait trials. The surface EMG signals were acquired at a sampling rate of 1926 samples/s, with a bandwidth ranging from 20 to 450 Hz, enabling high-fidelity recording of muscle activation patterns during dynamic activities. The four-channel FSR system, integrated into a customized wearable sandal, was used to capture localized plantar pressure values beneath the heel, midfoot, and toe regions. Individual adjustments were made to the sandal structure for each participant to ensure the proper alignment of the FSR membranes with anatomical landmarks of the foot (see Figure 5).

The FSR sensor unit had a body size of 27 × 46 × 13 mm and a mass of 19 g. The pressure values from FSR channel 1 (heel) were sampled at 1926 samples/s, while FSR channels 2–4 (midfoot and forefoot regions) were sampled at 148 samples/s, all within a consistent 50 Hz bandwidth. Though hardware dependent, this mixed sampling configuration allowed sufficient temporal resolution for initial contact and push off phase analysis.

EMG and FSR data streams were wirelessly transmitted to a workstation via EMGworks Acquisition v4.8.0 software (Delsys Inc., USA), enabling real-time synchronization and continuous monitoring throughout the experiments. The synchronized acquisition of neuromuscular and plantar pressure signals enabled the precise identification of gait events. It provided the foundational dataset for developing the EMG-driven GRU model discussed in this study.

### 3.3. Experimental Procedure

To collect reliable, multi-modal gait data for plantar pressure prediction, each participant was asked to walk at a natural, self-selected pace along a fixed 12 m straight walkway within a controlled indoor environment. The walkway surface was clean, level, and free of obstacles to minimize environmental influence on gait dynamics. Participants completed five walking trials, with brief 30 s rest intervals between trials to prevent muscle fatigue while preserving consistent physical conditions across sessions.

To ensure symmetry and natural gait, all participants wore a pair of customized sandals during the trials. Only the right sandal, however, was instrumented with a 4-channel FSR system integrated into the insole to capture localized plantar pressure values at the heel, midfoot, and toe regions. The left sandal was identical in shape and material but non-instrumented, ensuring that footwear conditions were consistent without introducing functional asymmetry. Each right sandal was individually fitted to align the FSR membranes precisely with anatomical pressure zones, enhancing the reliability of pressure readings during gait.

Each trial began with the right foot and ended at the 12 m mark, also stepping with the right foot to ensure uniformity in stride segmentation. While walking, the synchronized EMG and FSR systems recorded neuromuscular activation and plantar pressure distributions across complete gait cycles. This dual-sensor setup enabled the precise identification of key gait events, specifically HS, MST, and TO. These events were selected as focal points for pressure prediction due to their biomechanical relevance and functional applicability in AAFO control.

After each walking session, raw signals were reviewed to verify signal integrity. Any trials affected by sensor detachment, excessive noise, or motion artifacts were excluded from further analysis. The resulting dataset provided high-resolution EMG and pressure profiles suitable for training and validating the proposed GRU-based model to predict plantar pressure values from EMG features. This model is intended to inform future real-time gait phase recognition and event-triggered actuation in active ankle–foot orthoses.

### 3.4. Data Filtering and Preparation

The raw EMG and FSR signals underwent preprocessing to enhance signal clarity and enable accurate temporal alignment for model development. All processing steps were implemented using a custom Python-based script (Python 3.12). The EMG signals, recorded at 1926 Hz with a 20–450 Hz bandwidth, were filtered using a band pass filter to remove motion artifacts and high-frequency noise. A 50 Hz notch filter was applied to eliminate power line interference. The signals were then rectified and smoothed to generate clear EMG envelopes, which were normalized to reduce variability between subjects and prepare the data for biomechanical interpretation and machine learning input.

The FSR signals were processed based on their respective sampling rates. A low-pass filter (cutoff at 20 Hz) and baseline correction were applied to reduce noise and highlight foot–ground interactions. The pressure signals were then normalized between 0 and 1 to align with the EMG feature scale and ensure compatibility for training the GRU model. The resulting preprocessed datasets were time-aligned and segmented according to gait cycles, forming a reliable foundation for the subsequent biomechanical signal comparisons and the EMG-based pressure prediction model introduced in the following sections.

### 3.5. Biomechanical Signal Compatibility Evaluation

**Figure 6:**
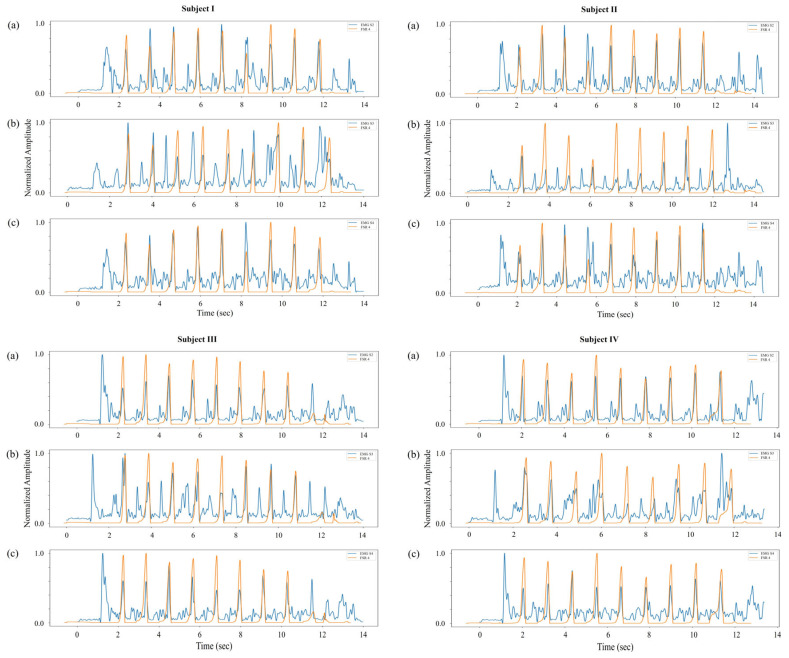
Comparison of representative normalized EMG envelopes (blue) with interpolated FSR channel 4 (toe region) outputs (orange) for subjects I–IV: (a) EMG sensor 2 positioned over the FL; (b) EMG sensor 3 over the SOL; (c) EMG sensor 4 over the GA muscle.

The suitability of surface EMG and FSR signals for capturing physiologically relevant ankle joint behavior was evaluated across three key movement types: plantarflexion, dorsiflexion, and inversion. This simple analysis confirmed that the acquired neuromuscular and mechanical signals reflect well-aligned, phase-specific activity consistent with expected gait biomechanics, establishing a reliable foundation for pressure estimation modeling. During plantarflexion, EMG activity from the FL, SOL, and GA muscles (sensors 2–4) was compared with data from FSR channel 4, located at the toe region. Due to the inherent delays between muscle activation and force output, a cross-correlation-based approach was used to determine the EMD between each EMG signal and the corresponding FSR segment. This alignment process enabled the precise synchronization of neuromuscular and mechanical data. After temporal alignment, the GA muscle (sensor 4) exhibited the most prominent and sharply timed activation peaks relative to toe pressure maxima (see Figure 6), reinforcing its critical role in generating plantarflexion force during the TO event. The FL and SOL muscles also displayed well-aligned activation patterns, consistent with their lateral stabilization and postural support functions.

**Figure 7:**
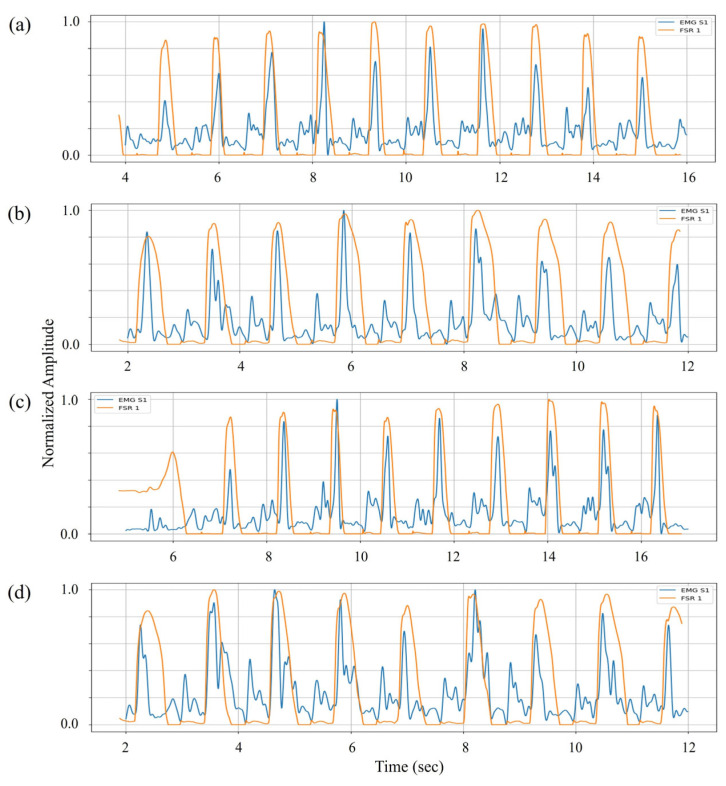
Comparison of normalized EMG signals (blue) from sensor 1 placed over the TA muscle with interpolated FSR channel 1 outputs (orange) from the heel region for dorsiflexion analysis. Subplots (**a**–**d**) correspond to subjects I–IV, respectively.

In the case of dorsiflexion, EMG signals from the TA muscle (sensor 1) were compared with FSR channel 1, positioned beneath the heel. The analysis revealed distinct EMG amplitude increases immediately preceding the heel pressure decline during the HO transition (see Figure 7). This activation reflects the initiation of dorsiflexion to lift the foot into the swing.

**Figure 8:**
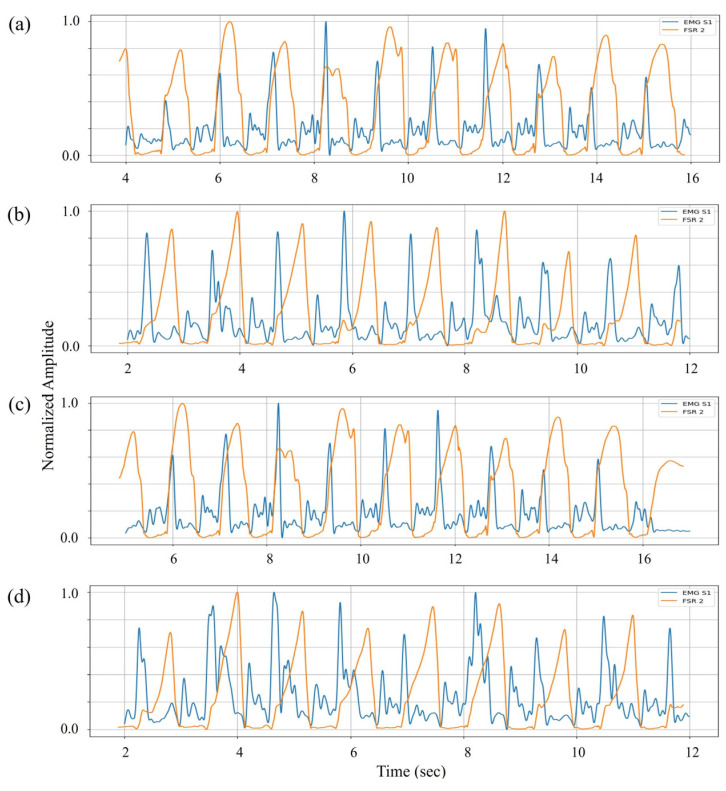
Comparison of normalized EMG signals (blue) from sensor 1 placed over the TA muscle with interpolated FSR channel 2 outputs (orange) from the midfoot region for inversion analysis. Subplots (**a**–**d**) correspond to subjects I–IV, respectively.

The EMD estimated in the previous phase was subsequently applied to time align the TA activity with midfoot pressure data for inversion analysis based on the shared functional involvement of the TA muscle in both motions. EMG sensor 1 was analyzed with FSR channel 2, which monitors midfoot pressure for inversion. Using the EMD from the dorsiflexion comparison, the signals were synchronized to isolate inversion-related activity. Following the primary dorsiflexion-associated activation, a secondary EMG activation pattern emerged during mid-to-late stance, aligned with rising pressure in the midfoot region (see Figure 8). This secondary activity reflects the TA muscle’s role in medial foot stabilization, contributing to inversion control as the body transitions through the stance phase. Despite inter-subject variability in signal morphology, the observed activation–pressure relationship remained consistent across all four participants.

Overall, these evaluations demonstrate precise and repeatable neuromechanical relationships between EMG signals and plantar pressure across the three movement types. The use of EMD-based alignment, particularly the pairing of sensor 4 and FSR channel 4 for plantarflexion calibration, proved essential in establishing temporal compatibility between muscle activation and ground contact forces (e.g., given that the TO event, as represented in Figure 2, is commonly associated with the point of maximum plantarflexion during the gait cycle). These results affirm the validity of the recorded signals and justify their integration into the EMG-based GRU model for real-time plantar pressure estimation introduced in the subsequent section.

## 4. Predictive Model Development

### 4.1. Model Architecture

The primary objective of this study is to design and implement a machine learning model capable of predicting plantar pressure values captured via FSR sensors based on time series EMG signals. These signals reflect the electrical activity of the muscles surrounding the ankle joint and are inherently temporal. Given the dynamic structure of EMG signals and the need to learn sequential dependencies across time, a Recurrent Neural Network (RNN) approach was adopted. Specifically, a GRU-based architecture was employed due to its ability to effectively capture long-term dependencies with reduced computational complexity compared with LSTM networks. The predictive pipeline integrated several key components: rigorous signal preprocessing, a sliding window segmentation strategy, focused feature extraction using RMS values, and an optimized GRU model trained to output synchronized FSR pressures corresponding to the heel, midfoot, and toe regions. This model contributes to the broader objective of neuromechanical modeling for real-time applications in assistive technologies such as AAFO, as discussed previously.

The final GRU-based model architecture was designed to efficiently process temporally segmented EMG data and produce multi-channel FSR output predictions. The model comprised the following layers:

- Input Layer: Accepts sequences of RMS values extracted from sliding windows of EMG data.
- GRU Layer: Includes 64 units with ‘return_sequences = True’ to preserve the temporal resolution across all time steps, ReLU activation was subsequently applied to the GRU outputs to introduce non-linearity.
- Dropout Layer: Applied with a rate of 0.4 to prevent overfitting by randomly disabling 40% of neurons during training.
- Dense Layer: A fully connected output layer with four units corresponding to the four FSR channels.
- Optimizer: The Adam optimizer with a learning rate of 0.0005 was selected for its adaptive learning rate capabilities and robust convergence behavior.

**Figure 9:**
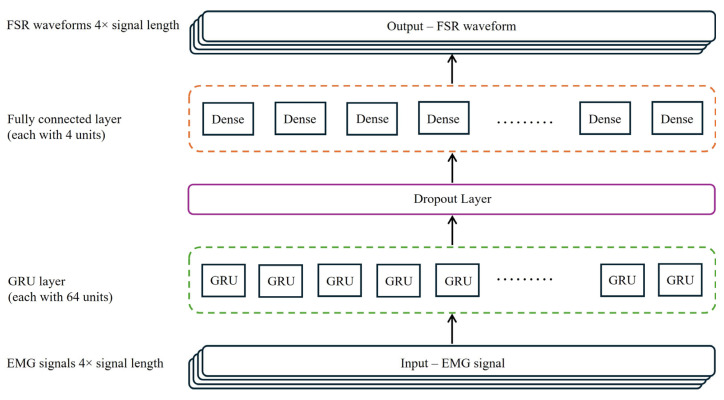
Architecture of the GRU-based predictive model.

The overall architecture of the proposed GRU-based predictive model, designed to map EMG signal features to FSR pressure outputs, is depicted in Figure 9. A detailed summary of the layer configuration and parameter distribution is provided in Table 1. The model comprises a total of 13,700 trainable parameters with no non-trainable parameters. This compact and efficient structure balances learning capacity with computational demands, facilitating real-time application potential for gait event prediction and control.

### 4.2. Model Training

The model was trained on a dataset comprising synchronized EMG and FSR signals recorded across multiple walking trials from different subjects. A total of 200 training epochs were used, with a batch size of 1 to preserve sequence integrity. The MSE was employed as the primary loss function, and the MAE served as the evaluation metric. Noisy or corrupted trials were excluded to maintain training quality. Additionally, subject V, who met the same inclusion criteria and demographic profile as the primary cohort, was included for further validation through multiple test cases, demonstrating the model’s robustness in handling inter-subject variability.

The outcomes of the training and validation processes are presented in the following section, highlighting the model’s predictive performance and generalization capabilities across subjects.

## 5. Results

**Figure 10:**
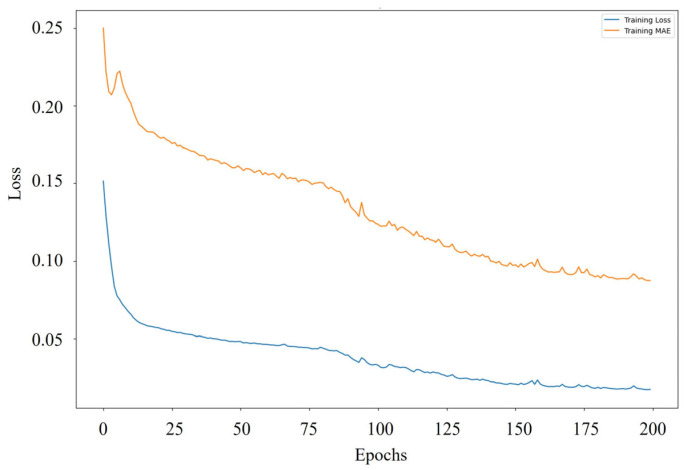
Training loss MSE (blue) and MAE (orange) trends over 200 epochs, indicating stable model convergence.

During training, the model achieved a low MSE of 0.0162 and an MAE of 0.0848, indicating a strong ability to map EMG-derived RMS features to FSR pressure values with minimal deviation. These results demonstrate that the model effectively captured the underlying neuromechanical relationships between muscle activation patterns and plantar pressure distributions throughout the gait cycle. Figure 10 depicts the evolution of the training loss MSE and MAE across 200 epochs. Both metrics show a clear downward trend, reflecting stable model convergence without signs of overfitting. Notably, the MAE curve exhibits a smooth decline and plateau behavior after approximately 150 epochs, suggesting that the model successfully optimized its internal parameters to achieve consistent predictive performance.

**Figure 11:**
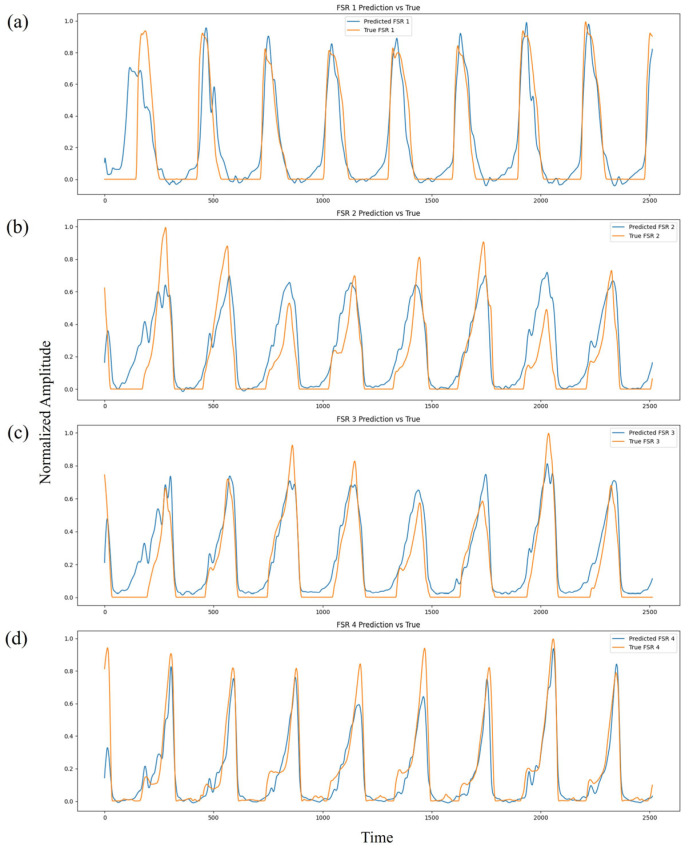
Comparison of predicted (blue) and ground-truth (orange) FSR waveforms across channels 1–4 (**a**–**d**), demonstrating accurate plantar pressure prediction over multiple gait cycles.

Figure 11 presents the comparison between predicted and ground-truth FSR pressure waveforms across the four sensing regions (heel, midfoot medial, midfoot lateral, and toe). The normalized pressure profiles reveal a high degree of temporal and amplitude alignment across multiple gait cycles. The predicted waveforms successfully reproduce major mechanical events such as HS, MST, and TO, demonstrating strong temporal fidelity. Minor discrepancies, primarily during rapid transition phases, are expected due to physiological variability, ambient noise, and the inherent electromechanical delay between neural activation and mechanical force output.

**Figure 12:**
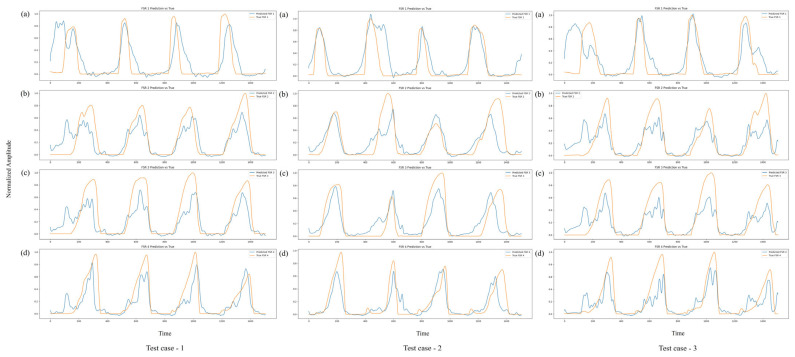
Comparison of predicted (blue) and actual (orange) FSR signals for subject V across three independent test cases. FSR channels 1–4 (a–d).

The evaluation of the test dataset, which consisted of unseen walking trials from subject V, who met the same inclusion criteria and demographic profile as the primary cohort, resulted in a comparable MSE of 0.0171 and MAE of 0.0832. The close alignment between training and test errors confirms that the model generalized effectively beyond the training data, maintaining predictive accuracy across the different individuals. This is particularly notable given the well-known variability in EMG signals associated with factors such as muscle morphology, activation strategies, and gait kinematics across subjects. Figure 12 further illustrates the model’s predictive performance on three independent walking trials from subject V (test cases 1–3).

Across all four FSR channels, the model consistently tracks the pressure dynamics over several gait cycles, capturing both the timing and amplitude of critical events. In particular, the model accurately reproduces pressure peaks associated with toe off (maximum plantarflexion) and midfoot loading during MST. Although minor amplitude mismatches are observed during certain transitions, these deviations predominantly occur during rapid loading and unloading phases, where dynamic changes in ground reaction forces are most pronounced. In the context of gait analysis, the loading phase corresponds to the period immediately following HS, when body weight is transferred onto the foot, while the unloading phase represents the progressive reduction in ground contact forces as the foot prepares for TO and transitions into the swing phase.

Despite these slight deviations, the overall temporal patterns and pressure dynamics remain accurately preserved, demonstrating the model’s ability to maintain biomechanical relevance even under inter-subject testing conditions.

## 6. Discussion

This study is positioned within the broader domain of developing an intelligent and adaptive AAFO system capable of delivering phase-specific, real-time assistance across multiple planes of ankle motion. To facilitate such functionality, we investigated the viability of leveraging neuromuscular signals, specifically surface EMG as input features for the prediction of spatially distributed plantar pressure patterns via a temporally responsive deep learning framework. The primary research objective was to determine whether temporal dependencies encoded within multi-sensory EMG signals could be effectively exploited to estimate continuous plantar loading profiles across anatomically defined foot regions during locomotion. We hypothesized that a GRU-based architecture, trained on systematically preprocessed EMG signals, could record physiologically meaningful neuromechanical associations under controlled experimental conditions. Although inter-subject variability in EMD was observed, the proposed model exhibited stable predictive performance across multiple healthy participants, indicating its generalization potential within normative populations. Ultimately, the proposed framework is intended to inform the control logic of next-generation AAFO by overcoming the limitations inherent to threshold-based or rule-based approaches, thereby enabling the fine-grained, data-driven modulation of orthotic support in response to the dynamic demands of human gait.

### 6.1. Predictive Model Performance and Generalization

The results obtained in this study highlight the feasibility and effectiveness of employing a GRU-based deep learning approach to predict plantar pressure distributions from EMG signals. This work followed a structured methodology, starting from biomechanical signal validation to predictive model development and evaluation, ensuring that the approach remained both technically sound and meaningful. Prior to model development, a Biomechanical Signal Compatibility Evaluation was performed to assess whether surface EMG signals from key ankle-related muscles appropriately reflected corresponding plantar pressure changes during major ankle joint movements. Through cross-correlation-based alignment techniques, it was confirmed that muscle activation patterns could reliably predict pressure variations related to plantarflexion, dorsiflexion, and inversion across different gait phases. This preliminary analysis provided strong justification for utilizing EMG signals as predictors of foot–ground interaction forces.

Building upon this foundation, a compact GRU-based predictive model was developed. The model utilized RMS values extracted from sliding windows of EMG signals, preserving temporal dependencies while simplifying feature space complexity. During training, the model achieved a low MSE of 0.0162 and an MAE of 0.0848, demonstrating the ability to learn robust mappings between neuromuscular activity and plantar pressure outputs. Evaluation on unseen walking trials from subject V, who met the same inclusion criteria as the primary cohort, resulted in comparably low errors (MSE = 0.0171, MAE = 0.0832), confirming the model’s generalization capability across individuals. The temporal patterns of predicted FSR signals closely tracked ground-truth measurements, effectively capturing key gait events such as HS, MST, and TO. Minor deviations were observed during rapid loading and unloading transitions, which are common sources of variability due to the dynamic nature of gait and inherent EMD.

### 6.2. Electromechanical Delay and Timing Alignment

A notable technical consideration in this study involves the EMD observed between muscle activation (EMG) and the resulting mechanical response (FSR). Cross-correlation analysis revealed subject-specific time lags for each EMG sensor, summarized in Table 2:

The observed EMD values, ranging approximately from −0.096 to −0.687 s (−96 to −687 ms), indicate that muscle activation (EMG) consistently preceded the mechanical response (FSR) across the subjects, aligning with expected neuromechanical timing. These values are physiologically reasonable for dynamic lower limb movements. Previous studies have reported that EMD typically ranges from approximately 30 to 200 ms during simple tasks such as isometric contractions, whereas longer delays up to 500–600 ms have been observed during complex dynamic activities like human gait [60,61]. Therefore, the delays found in this study align well with known physiological norms, particularly considering the multi-muscle, multi-phase characteristics of standard walking. While these variations are within expected ranges, they also suggest that a fixed time alignment approach may not fully account for individual neuromuscular differences. Future refinements could involve subject-specific dynamic alignment strategies to enhance prediction accuracy, especially in personalized orthotic device applications. Despite these distinctions, the model demonstrated consistently strong predictive performance across multiple subjects and trials. The relatively low MAE values indicate that predicted plantar pressures remained within acceptable error margins, ensuring that key gait transitions such as loading and unloading phases were accurately detected.

### 6.3. Predictive Robustness and Future Applicability

While the model’s predictive accuracy was validated within the current subject cohort, several methodological factors also suggest a strong potential for generalization to future data. The preprocessing steps, including band pass filtering (20–450 Hz), notch filtering, rectification, and RMS envelope extraction with a 10 Hz low-pass filter, standardized EMG signals and minimized subject-specific artifacts. The use of 200 sample sliding windows with 75% overlap produced over 50,000 sequences per subject, increasing temporal variability in the training data. The GRU architecture, with approximately 13,000 trainable parameters and 40% dropout, further reduced the overfitting risk. Notably, the model’s receptive field (251 samples) accommodated the observed electromechanical delay variability (−96 to −687 ms), enabling robust timing alignment across the subjects. These design choices are expected to sustain prediction accuracy (MAE 0.08–0.10) even when applied to future datasets processed under similar conditions.

### 6.4. EMG Input Variability on Predictive Performance

The number of EMG sensors also influenced the model’s predictive accuracy. Using a single sensor limited the model’s ability to capture co-activation patterns, increasing error rates by approximately 35–40% due to difficulties in distinguishing synergistic and antagonistic muscle activity. In contrast, employing 3–4 sensors targeting key agonist–antagonist groups such as TA, FL, SOL, and GA provided sufficient information on muscle coordination while maintaining minimal redundancy, resulting in MAE values around 0.08. Adding more sensors (up to 6–8) offered modest further reductions in error (3–5%) when large and diverse training datasets were available. However, increasing the number of sensor inputs also raised the risk of overfitting and roughly doubled the training time and computational load. When more than 6 sensors were used, the small accuracy gains were often outweighed by increased susceptibility to crosstalk between muscle signals and variability in sensor placement. Based on these considerations, the 4-sensor configuration adopted in this study provided a practical balance between predictive accuracy and model complexity.

### 6.5. Limitations and Future Directions

While the GRU-based model demonstrated consistent accuracy across the trials and the subjects, several important limitations should be acknowledged. First, the model was developed using data collected exclusively from four healthy male participants, with a fifth subject used for external validation. While this cohort allowed for controlled experimentation and cross-subject testing, the small sample size limits the statistical and clinical generalizability of the findings. Future work should extend the dataset to include a more diverse population, particularly individuals with neuromuscular disorders or pathological gait patterns. Such populations often exhibit irregular muscle activation profiles and altered gait timing, providing essential test cases for evaluating the robustness and clinical applicability of the proposed predictive model.

A second notable constraint involves the reliance on manual signal alignment via cross-correlation to synchronize EMG and FSR data. Although this method enabled the clear identification of subject-specific EMDs and ensured repeatable alignment during initial feasibility testing, it assumes fixed timing across all gait cycles. This limits its adaptability to intra-subject gait variability. In real-world conditions, especially in patients with impaired gait, these factors can introduce temporal misalignments that degrade prediction accuracy. Addressing this issue is critical for deploying EMG-driven models in dynamic, real-time control applications. To overcome this limitation, future studies will incorporate automated, subject-adaptive alignment strategies. Techniques such as dynamic time warping (DTW) can be used to non-linearly align EMG and pressure sequences on a cycle-by-cycle basis, keeping biomechanical phase relationships even under varying timing conditions. Additionally, delay-aware neural network architectures, such as GRUs or LSTMs enhanced with time shift embeddings, may allow the model to learn and compensate for timing mismatches directly from the data. These approaches would enable more robust and generalizable alignments, facilitating reliable real-time prediction across varying gait styles and subject populations.

Another methodological limitation is the exclusive use of surface EMG signals which, while informative, only reflect muscle activation but not limb orientation or joint kinematics. Future extensions may integrate IMUs or joint angle data to enhance the spatial understanding of foot–ground interactions, particularly on uneven terrain or in turning maneuvers. A multimodal sensor fusion approach could provide more comprehensive inputs for gait phase classification and orthotic control logic.

The number of EMG inputs required for the accurate prediction of pressure also warrants consideration. While our results showed that using three to four EMG channels offered a balance between prediction accuracy and model complexity, reducing the inputs to only one or two muscles led to a substantial increase in prediction error. This indicates that individual muscles provide distinct and non-redundant information. At the same time, the marginal improvement in accuracy beyond four sensors suggests that some of the neuromuscular coordination can be captured with a minimal sensor set. Future research may explore dimensionality reduction techniques, such as principal component analysis or feature selection algorithms, to identify a compact yet effective combination of muscle inputs. This line of investigation could further support the development of simplified, low-power, and wearable orthotic control systems.

Finally, this study’s design focused on offline prediction. While the current architecture is compact and optimized for real-time deployment, it has not yet been validated in live control scenarios. Future work will implement the trained model in embedded platforms and evaluate its real-time inference performance, latency, and responsiveness when integrated with active orthotic hardware.

In conclusion, this work presents a compact and computationally efficient GRU-based framework that enables the accurate estimation of plantar loading distributions from surface EMG signals. The model demonstrated strong predictive performance, capturing muscle-activation-to-pressure dynamics with low error margins across multiple gait cycles and test subjects. A four-sensor EMG configuration was shown to offer a pragmatic trade off between accuracy and system complexity, underscoring the potential for wearable implementation. Importantly, the framework lays the foundation for future AAFO control strategies aimed at restoring gait function, particularly in populations affected by neuromuscular impairments. Although the validation was limited to healthy young adults, the findings suggest translational potential in older individuals with mobility challenges, where adaptive orthotic control based on real-time muscle activation could improve gait stability and reduce fall risk. Further investigations involving clinical populations and real-time deployment will be essential to realize these applications.

## Figures and Tables

**Table 1:** Summary of model architecture and parameter distribution.

Layer (Type)	Output Shape	No. of Parameters	Description
GRU (64 units)	(None, 2513, 64)	13,440	Sequential feature extraction and temporal dependency modeling
Dropout (rate = 0.4)	(None, 2513, 64)	0	Regularization to prevent overfitting
Dense (4 units)	(None, 2513, 4)	260	Output layer for predicting four FSR pressure values

**Table 2:** Subject-specific EMD values (seconds) between EMG signals and FSR outputs.

Subject	EMG Sensor 1	EMG Sensor 2	EMG Sensor 3	EMG Sensor 4
I	−0.108	−0.626	−0.112	−0.623
II	−0.146	−0.590	—	−0.636
III	−0.124	−0.607	−0.096	−0.631
IV	−0.156	−0.686	−0.673	−0.687

## Data Availability

The data presented in this study are available on request.

## Abbreviations

The following abbreviations are used in this manuscript:

AAFO	active ankle–foot orthoses
AFO	ankle–foot orthoses
DTW	dynamic time warping
EMD	electromechanical delay
EMG	electromyography
FF	foot flat
FL	fibularis longus
FSR	force-sensitive resistor
GA	gastrocnemius
GRU	Gated Recurrent Unit
HO	heel off
HS	heel strike
IMU	inertial measurement unit
LSTM	long short-term memory
MAE	mean absolute error
MLP	multilayer perceptron
MPC	model predictive control
MSW	mid-swing
MSE	mean squared error
MST	mid stance
PS	pre-swing
RMS	root mean square
SOL	soleus
TA	tibialis anterior
TO	toe off

## References

[1] Cabinet Office, Japan Annual Report on the Ageing Society [Summary] FY2024. https://www8.cao.go.jp/kourei/english/annualreport/2024/pdf/2024.pdf.

[2] Tinetti M.E., Speechley M., Ginter S.F. (1988). Risk Factors for Falls among Elderly Persons Living in the Community. N. Engl. J. Med..

[3] Perry J., Burnfield J.M. (2010). Gait Analysis: Normal and Pathological Function.

[4] Wren T.A.L., Gorton G.E., Õunpuu S., Tucker C.A. (2011). Efficacy of Clinical Gait Analysis: A Systematic Review. Gait Posture.

[5] Leardini A., O’Connor J.J., Giannini S. (2014). Biomechanics of the Natural, Arthritic, and Replaced Human Ankle Joint. J. Foot Ankle Res..

[6] Bregman D.J.J., van der Krogt M.M., de Groot V., Harlaar J., Wisse M., Collins S.H. (2011). The Effect of Ankle Foot Orthosis Stiffness on the Energy Cost of Walking: A Simulation Study. Clin. Biomech..

[7] Neptune R.R., Wright I.C., van den Bogert A.J. (1999). Muscle Coordination and Function during Cutting Movements. Med. Sci. Sports Exerc..

[8] Ippersiel P., Robbins S.M., Dixon P.C. (2021). Lower-Limb Coordination and Variability during Gait: The Effects of Age and Walking Surface. Gait Posture.

[9] Gunaratne P.N., Tamura H. (2024). A Review: Developments in Hardware Systems of Active Ankle Orthoses. Sensors.

[10] Esquenazi A., Talaty M., Packel A., Saulino M. (2012). The ReWalk Powered Exoskeleton to Restore Ambulatory Function to Individuals with Thoracic-Level Motor-Complete Spinal Cord Injury. Am. J. Phys. Med. Rehabil..

[11] Veneman J.F., Kruidhof R., Hekman E.E.G., Ekkelenkamp R., Van Asseldonk E.H.F., van der Kooij H. (2007). Design and Evaluation of the LOPES Exoskeleton Robot for Interactive Gait Rehabilitation. IEEE Trans. Neural Syst. Rehabil. Eng..

[12] Maqbool H.F., Husman M.A.B., Awad M.I., Abouhossein A., Mehryar P., Iqbal N., Dehghani-Sanij A.A. Real-Time Gait Event Detection for Lower Limb Amputees Using a Single Wearable Sensor. Proceedings of the 2016 38th Annual International Conference of the IEEE Engineering in Medicine and Biology Society (EMBC).

[13] Fatone S., Owen E., Gao F., Shippen G., Orendurff M.S., Bjornson K. (2021). Comparison of Sagittal Plane Stiffness of Nonarticulated Pediatric Ankle-Foot Orthoses Designed to Be Rigid. JPO J. Prosthet. Orthot..

[14] Jiménez-Fabián R., Verlinden O. (2012). Review of Control Algorithms for Robotic Ankle Systems in Lower-Limb Orthoses, Prostheses, and Exoskeletons. Med. Eng. Phys..

[15] Shamaei K., Napolitano P.C., Dollar A.M. A Quasi-Passive Compliant Stance Control Knee-Ankle-Foot Orthosis. Proceedings of the 2013 IEEE 13th International Conference on Rehabilitation Robotics (ICORR).

[16] Sawicki G.S., Ferris D.P. (2008). Powered Ankle Exoskeletons Reveal the Metabolic Cost of Plantar Flexor Mechanical Work during Walking with Longer Steps at Constant Step Frequency. J. Exp. Biol..

[17] Tao W., Liu T., Zheng R., Feng H. (2012). Gait Analysis Using Wearable Sensors. Sensors.

[18] Chen B., Ma H., Qin L.-Y., Gao F., Chan K.-M., Law S.-W., Qin L., Liao W.-H. (2016). Recent Developments and Challenges of Lower Extremity Exoskeletons. J. Orthop. Transl..

[19] Gao Y., Zheng J., Yang C., Yan R., Wang C., Tang J., Jiang Z. (2025). Real-Time Gait Phase Detection Based on LSTM-ResMLP-LightGBM Approach for Exoskeleton in Outdoor Activity. IEEE Access.

[20] Panizzolo F.A., Galiana I., Asbeck A.T., Siviy C., Schmidt K., Holt K.G., Walsh C.J. (2016). A Biologically-Inspired Multi-Joint Soft Exosuit That Can Reduce the Energy Cost of Loaded Walking. J. Neuroeng. Rehabil..

[21] Jamali A., Abdul Razak A.S., Mohamaddan S. (2025). Imposing Neural Networks and PSO Optimization in the Quest for Optimal Ankle-Foot Orthosis Dynamic Modelling. TELKOMNIKA Telecommun. Comput. Electron. Control.

[22] Jamali A., Abdul Razak A.S., Mohamaddan S. An In-Depth Study of Ankle-Foot Orthosis Dynamics Modeling: Leveraging Non-Parametric Approach via Artificial Neural Networks. Proceedings of the 2023 IEEE 9th International Conference on Smart Instrumentation, Measurement and Applications (ICSIMA).

[23] Shefa F.R., Sifat F.H., Uddin J., Ahmad Z., Kim J.-M., Kibria M.G. (2024). Deep Learning and IoT-Based Ankle–Foot Orthosis for Enhanced Gait Optimization. Healthcare.

[24] Shefa F.R., Sifat F.H., Shah S.C., Kibria M.G. IoT-Based Smart Ankle-Foot Orthosis for Patients with Gait Imbalance. Proceedings of the 2023 23rd International Conference on Control, Automation and Systems (ICCAS).

[25] Ulkir O., Akgun G., Nasab A., Kaplanoglu E. (2021). Data-Driven Predictive Control of a Pneumatic Ankle Foot Orthosis. Adv. Electr. Comput. Eng..

[26] DeBoer B., Hosseini A., Rossa C. (2023). Model Predictive Control of an Active Ankle-Foot Orthosis with Non-Linear Actuation Constraints. Control Eng. Pract..

[27] Jun K., Lee S., Lee D.-W., Kim M.S. (2021). Deep Learning-Based Multimodal Abnormal Gait Classification Using a 3D Skeleton and Plantar Foot Pressure. IEEE Access.

[28] Phinyomark A., Khushaba R.N., Scheme E. (2018). Feature Extraction and Selection for Myoelectric Control Based on Wearable EMG Sensors. Sensors.

[29] Hudgins B., Parker P., Scott R.N. (1993). A New Strategy for Multifunction Myoelectric Control. IEEE Trans. Biomed. Eng..

[30] Cho K., van Merriënboer B., Gulcehre C., Bahdanau D., Bougares F., Schwenk H., Bengio Y. (2014). Learning Phrase Representations Using RNN Encoder-Decoder for Statistical Machine Translation. arXiv.

[31] Saponas T.S., Tan D.S., Morris D., Balakrishnan R., Turner J., Landay J.A. Enabling Always-Available Input with Muscle-Computer Interfaces. Proceedings of the UIST ’09: Proceedings of the 22nd Annual ACM Symposium on User Interface Software and Technology.

[32] Vrieling A.H., van Keeken H.G., Schoppen T., Otten E., Halbertsma J.P.K., Hof A.L., Postema K. (2008). Gait Initiation in Lower Limb Amputees. Gait Posture.

[33] Hoover C.D., Fulk G.D., Fite K.B. (2013). Stair Ascent with a Powered Transfemoral Prosthesis under Direct Myoelectric Control. IEEE/ASME Trans. Mechatron..

[34] Whittle M. (2007). Gait Analysis: An Introduction.

[35] Winter D.A. (2009). Biomechanics and Motor Control of Human Movement.

[36] Nigg B.M. (2006). Biomechanics of the Musculo-Skeletal System.

[37] MacKinnon C.D., Winter D.A. (1993). Control of Whole Body Balance in the Frontal Plane during Human Walking. J. Biomech..

[38] Mueller M.J., Sinacore D.R., Hoogstrate S., Daly L. (1994). Hip and Ankle Walking Strategies: Effect on Peak Plantar Pressures and Implications for Neuropathic Ulceration. Arch. Phys. Med. Rehabil..

[39] Dugan S.A., Bhat K.P. (2005). Biomechanics and Analysis of Running Gait. Phys. Med. Rehabil. Clin. N. Am..

[40] Kulmala J.-P., Korhonen M.T., Kuitunen S., Suominen H., Heinonen A., Mikkola A., Avela J. (2016). Whole Body Frontal Plane Mechanics across Walking, Running, and Sprinting in Young and Older Adults. Scand. J. Med. Sci. Sports.

[41] Avers D., Wong R.A. (2020). Guccione’s Geriatric Physical Therapy.

[42] Liu J., Tan X., Jia X., Li T., Li W. (2024). A Gait Phase Recognition Method for Obstacle Crossing Based on Multi-Sensor Fusion. Sens. Actuators A Phys..

[43] Dollar A.M., Herr H. (2008). Lower Extremity Exoskeletons and Active Orthoses: Challenges and State-of-The-Art. IEEE Trans. Robot..

[44] Morbidoni C., Cucchiarelli A., Agostini V., Knaflitz M., Fioretti S., Di Nardo F. (2021). Machine-Learning-Based Prediction of Gait Events from EMG in Cerebral Palsy Children. IEEE Trans. Neural Syst. Rehabil. Eng..

[45] Dimitrov H., Bull J., Farina D. (2020). Real-Time Interface Algorithm for Ankle Kinematics and Stiffness from Electromyographic Signals. IEEE Trans. Neural Syst. Rehabil. Eng..

[46] Brockett C.L., Chapman G.J. (2016). Biomechanics of the Ankle. Orthop. Trauma.

[47] Lundberg A., Svensson O., Nemeth G., Selvik G. (1989). The Axis of Rotation of the Ankle Joint. J. Bone Jt. Surg. Br. Vol..

[48] Barnett C.H., Napier J.R. (1952). The Axis of Rotation at the Ankle Joint in Man; Its Influence upon the Form of the Talus and the Mobility of the Fibula. J. Anat..

[49] Manter J.T. (1941). Movements of the Subtalar and Transverse Tarsal Joints. Anat. Rec..

[50] Procter P., Paul J.P. (1982). Ankle Joint Biomechanics. J. Biomech..

[51] Hicks J.H. (1954). The Mechanics of the Foot. II. The Plantar Aponeurosis and the Arch. J. Anat..

[52] Kirby K. (2000). Biomechanics of the Normal and Abnormal Foot. J. Am. Podiatr. Med. Assoc..

[53] Moore K.L., Dalley A.F., Agur A.M.R. (2017). Clinically Oriented Anatomy.

[54] Hermens H.J., Freriks B., Disselhorst-Klug C., Rau G. (2000). Development of Recommendations for SEMG Sensors and Sensor Placement Procedures. J. Electromyogr. Kinesiol..

[55] Perotto A., Delagi E.F., Charles C. (2005). Anatomical Guide for the Electromyographer: The Limbs and Trunk.

[56] Winter D.A. (1991). The Biomechanics and Motor Control of Human Gait: Normal, Elderly and Pathological.

[57] Rhim H.C., Dhawan R., Gureck A.E., Lieberman D.E., Nolan D.C., Elshafey R., Tenforde A.S. (2022). Characteristics and Future Direction of Tibialis Posterior Tendinopathy Research: A Scoping Review. Medicina.

[58] Clancy E.A., Morin E.L., Merletti R. (2002). Sampling, Noise-Reduction and Amplitude Estimation Issues in Surface Electromyography. J. Electromyogr. Kinesiol..

[59] Farina D., Merletti R., Enoka R.M. (2004). The Extraction of Neural Strategies from the Surface EMG. J. Appl. Physiol..

[60] Cavanagh P.R., Komi P.V. (1979). Electromechanical Delay in Human Skeletal Muscle under Concentric and Eccentric Contractions. Eur. J. Appl. Physiol. Occup. Physiol..

[61] Norman R.W., Komi P.V. (1979). Electromechanical Delay in Skeletal Muscle under Normal Movement Conditions. Acta Physiol. Scand..

